# Independent Risk Factors of Non-Resolution Mural Thrombus in ST-Segment Elevation Myocardial Infarction Patients with Left Ventricular Aneurysms

**DOI:** 10.31083/RCM28222

**Published:** 2025-05-08

**Authors:** Meng Wang, Mengwan Li, Wenheng Liu, Jian Li, Dan Chen, Ziqing Wang, Qilong Guo, Shouling Mi, Junhua Ge

**Affiliations:** ^1^Department of Cardiology, The Affiliated Hospital of Qingdao University, Qingdao Municipal Key Laboratory of Hypertension (Key Laboratory of Cardiovascular Medicine), 266000 Qingdao, Shandong, China; ^2^Department of Cardiology, Zhongshan Hospital, Fudan University, Shanghai Institute of Cardiovascular Diseases, National Clinical Research Center for Interventional Medicine, 200032 Shanghai, China

**Keywords:** left ventricular aneurysm, mural thrombus, risk factors, non-resolution, left ventricular aneurysm area, mural thrombus area

## Abstract

**Background:**

The clinical prognosis of ST-elevation myocardial infarction (STEMI) patients with mural thrombus in left ventricular aneurysm (MTLVA) remains poor; moreover, the risk factors associated with the non-resolution (persistent or recurrent) of MTLVA are not well understood. This study aimed to identify independent risk factors for MTLVA non-resolution.

**Methods:**

A total of 133 STEMI patients (mean age 62 ± 11 years, 80.5% male) with MTLVA, admitted to our department between 2014 and 2022, were included in this retrospective analysis. Patients were categorized into two groups: resolution (n = 59) and non-resolution [persistent (n = 72) or recurrent (n = 2) MTLVA; n = 74]. The median follow-up duration was 25 months, during which adverse events were monitored, including stroke, re-revascularization, major bleeding, systemic embolism, and cardiac death.

**Results:**

The prevalence of non-resolution was 55.6%. Non-resolution was significantly associated with elevated lipoprotein (a) [Lp(a)] levels (>270 mg/L, hazard ratios (HR) 2.270, *p* = 0.003), larger left ventricular aneurysm (LVA) area (>4.5 cm^2^, HR 4.038, *p* < 0.001), and greater mural thrombus (MT) area (>2.2 cm^2^, HR 2.40, *p* = 0.002), independent of other risk factors, such as hypercholesterolemia and left circumflex artery (LCX)-related STEMI. Baseline left ventricular ejection fraction (LVEF) was lower in the non-resolution group (41.7% vs. 45.7%, *p* = 0.008). During follow-up, the LVEF remained lower in the non-resolution group and increased in the resolution group. The composite of adverse events was significantly higher in the non-resolution group (28.4% vs. 8.5%, *p* = 0.003), including stroke (*p* = 0.025) and systemic embolism (*p* = 0.034).

**Conclusions:**

Independent risk factors for thrombus non-resolution in STEMI patients with MTLVA include elevated Lp(a), larger LVA and MT areas. These factors contribute to thrombus persistence and are associated with worse clinical outcomes. However, further studies are needed to assess targeted management strategies for high-risk patients.

## 1. Introduction

Current revascularization strategies and modern antithrombotic therapy have markedly reduced the incidence of left ventricular thrombus (LVT) over the last decades [1]. Contemporary data showed that the incidence of LVT might still be as high as 15% to 25% in patients with ST-elevation myocardial infarction (STEMI) post percutaneous coronary intervention (PCI) and modern antithrombotic therapy [2, 3]. Nowadays, mural thrombus (MT) in left ventricular aneurysm (LVA) post STEMI remains a clinical challenge associated with poor outcome [4, 5, 6].

Guidelines recommend antithrombotic drug treatment for least 3 months and up to 6 months depending on follow-up imaging results for MT in LVA (MTLVA) patients [7, 8]. Persistence or recurrence MTLVA remained as difficult clinical scenario despite modern antithrombotic therapy [5, 9, 10, 11]. Observational research suggests that persistence of LVT is not uncommon [12]. Lattuca *et al*. [5] found that total regression was only achieved in 62.3% patients with LVT within a median time of 103 days. Recently, Zhou *et al*. [4] demonstrated similar LVT resolution rate (63.7%) in another LVT patient cohort, during a median follow-up of 1.2 years after LVT resolution, LVT recurrence rate was 24.3% (n = 28) in this patient cohort. As expected, persistent or recurrent LVT were associated with even higher risk of major adverse cardiovascular events (MACE), embolic, or major bleeding complications, as well as mortality as compared to patients with complete LVT resolution [2, 4, 6, 7].

Identifying independent risk factors for non-resolution (persistent or recurrence) MTLVA is of clinical importance for risk stratification and decision-making for therapeutic and monitoring strategies. In this retrospective study, we explored the independent determinants of MTLVA non-resolution and compared the clinical outcomes in MTLVA patients with or without complete resolution.

## 2. Methods

### 2.1 Study Population and Data Collection 

We screened 303 consecutive STEMI patients with LVA who were admitted to our department between March 2014 and June 2022. Of these, transthoracic echocardiography (TTE) identified MT in 133 patients, leading to their classification as MTLVA cases. Two independent experts confirmed the diagnosis of MT through imaging review. These 133 patients were included in this retrospective clinical cohort study for further analysis. All patients received standard antithrombotic therapy in accordance with the relevant guidelines issued by the European Society of Cardiology (post-LVA) [8, 13]. Smoking status was defined as former or current smoking based on self-reported history. Alcohol intake was defined as an average daily intake exceeding 40 g for men or 20 g for women, sustained for at least five years. Patients were followed for a median duration of 25 months, during which clinical visit and echocardiographic examinations were conducted at each follow-up visit.

### 2.2 Detection of MT and LVA

Detection of MT and LVA was accomplished using TTE (Philips EPIQ-7C, Philips Healthcare, Amsterdam, The Netherlands). MT appeared as an echodense mass adjacent to the left ventricular (LV) wall, with confirmation of LVA achieved through akinetic or dyskinetic LV wall motion, which bulged outward during systole. To differentiate MT from the underlying myocardium, a distinct thrombus-blood interface was necessary, and MT had to be observable on at least two views throughout the cardiac cycle. Quantitative evaluation of LVA and MT characteristics was performed offline, involving measurements of the maximal long and short diameters of LVA (LVA_LD and LVA_SD), the area of LVA (LVA_area), the maximal long and short diameters of MT (MT_LD and MT_SD), and the area of MT (MT_area). All measurements were based on the mean of three assessments.

### 2.3 Grouping 

Patients were divided into two groups based on the resolution status of MTLVA. The MTLVA resolution group was characterized by the complete disappearance of MTLVA following antithrombotic treatment, as confirmed by echocardiographic examinations during follow-up. Conversely, the MTLVA non-resolution group comprised patients in whom MTLVA persisted or recurred despite antithrombotic treatment, as observed through echocardiographic assessment during follow-up.

### 2.4 Outcomes

The primary endpoint was defined as the occurrence of adverse events during follow-up, encompassing stroke, re-revascularization, major bleeding, systemic embolism, or cardiac death.

### 2.5 Statistical Analysis

Continuous variables are expressed as mean ± standard deviation or median (interquartile range, IQR), and categorical variables as number (percent). Group differences were compared using the independent samples *t*-test or Mann-Whitney *U* test, as appropriate. For categorical data, comparisons across groups were made using the Chi-square test or Fisher’s exact test. Dynamic changes in left ventricular ejection fraction (LVEF) over time were analyzed using one-way repeated measures analysis of variance (ANOVA) with the General Linear Model. Diagnostic performance of LVA and MT for predicting MTLVA non-resolution was assessed using receiver operating characteristic (ROC) curves, with a comparison among the areas under curves (AUC). Optimal cut-offs for observed variables such as laboratory parameters and echocardiographic parameters in this analysis were determined using the Youden Index method derived from ROC estimation. To identify independent risk factors associated with clinical outcomes, univariable and multivariable Cox proportional hazards regression analysis were performed. Variables with *p*-value < 0.05 in the univariable Cox analysis were included in the multivariable models, which were constructed using “backward stepwise” selection method. Collinearity was assessed using the correlation coefficient, with a threshold of >0.70 indicating the presence of significant collinearity between variables. To prevent multicollinearity, which could distort the estimation of regression coefficients and affect model stability, variables exhibiting collinearity were excluded from the multivariable models. Only independent variables with no significant collinearity were retained for inclusion. Results were reported as hazard ratios (HR) with corresponding 95% confidence intervals (CI). A *p* value < 0.05 (two-tailed test) was considered statistically significant. The statistical analysis was performed using the SPSS statistical software, version 28.0 (IBM SPSS Statistics, Chicago, IL, USA).

## 3. Results

### 3.1 Baseline Clinical and Echocardiographic Characteristics of STEMI Patients with MTLVA Resolution or Non-resolution 

All patients underwent serial echocardiography follow-up for MTLVA assessment, with a median of 4 (3–5) evaluations. Over a median follow-up of 25 (14–39) months, MTLVA resolution was observed in 44.4% (59 out of 133) of patients with STEMI, while 55.6% (74 out of 133) exhibited non-resolution including two cases of MTLVA recurrence. Specifically, MTLVA disappeared in 42 patients at the second echocardiography [median period of 46 (21–98) days], in 52 patients at the third echocardiography [median period of 6.5 (2.8–18.2) months], and in 59 patients at the fourth echocardiography [median period of 12.0 (8.5–23.5) months].

Table 1 details the baseline characteristics of STEMI patients with MTLVA resolution and non-resolution. The mean age was 62 ± 11 years, 107 (80.5%) patients were male. In comparison to patients with MTLVA resolution, those with MTLVA non-resolution showed a higher proportion of excessive alcohol drinkers (25.7% vs. 11.9%, *p* = 0.046) and elevated D-Dimer levels (median 720 vs. 435 ng/mL, *p* = 0.002).

**Table 1.** **Baseline clinical and echocardiographic characteristics in in STEMI patients with MTLVA resolution and non-resolution**.

			Total	MTLVA resolution	MTLVA non-resolution	*p* value
No. [n (%)]			133 (100)	59 (44.4)	74 (55.6)	
Age (years)			62 ± 11	63 ± 10	62 ± 12	0.811
Male [n (%)]			107 (80.5)	45 (76.3)	62 (83.8)	0.278
BMI (kg/m²)			24.7 ± 3.3	24.6 ± 3.4	24.8 ± 3.3	0.330
Hypertension [n (%)]			70 (52.6)	31 (52.5)	39 (52.7)	0.985
Diabetes mellitus [n (%)]			37 (27.8)	16 (27.1)	21 (28.4)	0.872
Hypercholesterolemia [n (%)]			50 (37.6)	18 (30.5)	32 (43.2)	0.132
Smoking [n (%)]			66 (49.6)	24 (40.7)	42 (56.8)	0.065
Alcohol intake [n (%)]			26 (19.5)	7 (11.9)	19 (25.7)	0.046
Laboratory data						
	WBC (10^9^/L)		7.48 (5.99–8.94)	7.58 (6.11–9.11)	7.29 (5.84–8.63)	0.375
	Hb (g/L)		140 (126–150)	137 (128–155)	141 (125–148)	0.747
	PLT (10^9^/L)		215 (182–267)	211 (174–260)	215 (184–273)	0.873
	D-Dimer (ng/mL)		530 (349–1007)	435 (297–687)	720 (400–1297)	0.002
	NT-proBNP (pg/mL)		890 (323–2680)	1077 (430–3094)	800 (300–2702)	0.346
	AST (U/L)		23.0 (15.1–44.0)	23.5 (17.0–53.5)	20.5 (16.0–35.0)	0.253
	ALT (U/L)		25.0 (19.0–40.0)	26.0 (15.3–41.0)	24.0 (15.0–49.0)	0.934
	Cr (mg/dL)		82.0 (64.0–99.0)	80.0 (63.0–95.7)	86.0 (64.0–103.0)	0.300
	TG (mmol/L)		1.24 (0.92–1.75)	1.28 (0.96–1.70)	1.17 (0.86–1.86)	0.803
	TC (mmol/L)		4.20 (3.41–4.85)	4.24 (3.45–4.95)	4.03 (3.39–4.84)	0.433
	LDL (mmol/L)		2.43 (1.88–2.97)	2.49 (1.90–3.00)	2.40 (1.80–2.91)	0.600
	Uric acid (µmol/L)		364 (290–427)	354 (285–408)	379 (293–442)	0.389
	Lp(a) (mg/L)		182 (111–359)	164 (79–269)	204 (118–409)	0.093
STEMI treatment [n (%)]						0.376
	PCI only		98 (73.7)	47 (79.7)	51 (68.9)	
	CABG only or PCI+CABG		26 (19.5)	9 (15.3)	17 (23.0)	
	Thrombolytic administration only		9 (6.8)	3 (5.1)	6 (8.1)	
	Involved coronary vessel					
		LAD	118 (88.7)	51 (86.4)	67 (90.5)	0.458
		LCX	50 (37.6)	16 (27.1)	34 (45.9)	0.026
		RCA	39 (29.3)	16 (27.1)	23 (31.1)	0.618
Antithrombotic Strategy Following MTLVA [n (%)]						
	Aspirin		103 (77.4)	45 (76.3)	58 (78.4)	0.773
	Clopidogrel		67 (50.4)	30 (50.8)	37 (50.0)	0.923
	Ticagrelor		23 (17.3)	11 (18.6)	12 (16.2)	0.713
	Indobufen		4 (3.0)	4 (6.8)	0 (0.0)	0.023
	VKAs		76 (57.1)	39 (66.1)	37 (50.0)	0.062
	NOACs		54 (40.6)	17 (28.8)	37 (50.0)	0.013
		Rivaroxaban	44 (33.1)	14 (23.7)	30 (40.5)	0.041
		Dabigatran	10 (7.5)	3 (5.1)	7 (9.5)	0.342
	Anticoagulation only		17 (12.8)	6 (10.2)	11 (14.9)	0.420
	Anticoagulation + antiplatelet therapy		35 (26.2)	16 (27.1)	19 (25.7)	0.851
	Anticoagulation + dual antiplatelet therapy		81 (60.9)	37 (62.7)	44 (59.5)	0.703
Cardiac related medications [n (%)]						
	ß-blockers		112 (84.2)	52 (88.1)	60 (8.1)	0.268
	ACEIs/ARBs		46 (34.6)	18 (30.5)	28 (37.8)	0.377
	ARNIs		35 (26.3)	21 (35.6)	14 (18.9)	0.030
	MRAs		86 (64.7)	37 (62.7)	49 (66.2)	0.674
	SGLT2i		8 (6.0)	2 (3.4)	6 (8.1)	0.300
	Loop diuretic		124 (93.2)	51 (86.4)	73 (98.6)	0.011
	CCB		33 (24.8)	19 (32.2)	14 (18.9)	0.078
	Nitrates		133 (100)	59 (100)	74 (100)	-
	Statins		133 (100)	59 (100)	74 (100)	-
Echocardiography						
	LVEF (%)		43.5 ± 8.8	45.7 ± 8.8	41.7 ± 8.5	0.008
	LVA_LD (cm)		3.68 ± 1.30	2.78 ± 0.56	4.40 ± 1.29	<0.001
	LVA_SD (cm)		2.31 ± 0.79	1.83 ± 0.55	2.70 ± 0.74	<0.001
	LVA_area (cm²)		5.65 (3.53–10.15)	3.53 (2.83–4.95)	8.89 (5.99–12.80)	<0.001
	MT_LD (cm)		2.44 ± 0.96	1.86 ± 0.53	2.91 ± 0.98	<0.001
	MT_SD (cm)		1.29 ± 0.57	0.99 ± 0.36	1.53 ± 0.59	<0.001
	MT_area (cm²)		2.16 (1.15–3.94)	1.34 (0.82–2.12)	3.56 (1.79–4.91)	<0.001
Outcomes [n (%)]						
	FUP duration (months)		20 (12–33)	25 (15–40)	16 (10–27)	
	Surgery for LVA		5 (3.8)	2 (3.4)	3 (4.1)	1.000
	CV death		7 (5.3)	1 (1.7)	6 (8.1)	0.132
	Major bleeding		7 (5.3)	1 (1.7)	6 (8.1)	0.132
	rPCI		1 (0.8)	1 (1.7)	0 (0.0)	0.444
	Stroke		13 (9.8)	2 (3.4)	11 (14.9)	0.027
	Systemic embolism		4 (3.0)	0 (0.0)	4 (5.4)	0.129
	Adverse events		26 (19.5)	5 (8.5)	21 (28.4)	0.004

ACEIs, angiotensin-converting enzyme inhibitors; ALT, alanine aminotransferase; ARNIs, angiotensin receptor neprilysin inhibitors; ARBs, angiotensin II receptor antagonists; AST, aspartate aminotransferase; BMI, body mass index; CABG, coronary artery bypass graft; CCB, calcium antagonist; Cr, creatinine; FUP, follow-up; Hb, hemoglobin; LAD, left anterior artery; LCX, left circumflex artery; LDL, low-density lipoprotein; Lp(a), lipoprotein(a); LVA, left ventricular aneurysm; LVA_LD, left ventricular aneurysm long diameter; LVA_SD, left ventricular aneurysm short diameter; LVEF, left ventricular ejection fraction; MRAs, mineralcorticoid recept antagonist; MT_LD, mural thrombus long diameter; MTLVA, mural thrombus in left ventricular aneurysm; MT_SD, mural thrombus short diameter; NOACs, non-vitamin K oral anticoagulants; NT-proBNP, N-terminal pro-brain natriuretic peptide; PCI, percutaneous coronary intervention; PLT, platelet; RCA, right coronary artery; SGLT2i, sodium-dependent glucose transporters 2 inhibitors; STEMI, ST-segment elevation myocardial infarction; TC, total cholesterol; TG, triglyceride; VKAs, vitamin K antagonists; WBC, white blood cell; CV, cardiovascular; rPCI, repeat percutaneous coronary intervention.

### 3.2 Antithrombotic Strategy

All patients received low molecular weight heparin at admission. Post-discharge, 57.1% (n = 76) were on vitamin K antagonists (VKAs), 33.1% (n = 44) on rivaroxaban, and 7.5% (n = 10) on dabigatran. Anticoagulation was not applied to three patients due to high bleeding risk. Concomitant antiplatelet therapy was common (87.2%, n = 116), with 26.2% (n = 35) on anticoagulation + single antiplatelet and 60.9% (n = 81) on anticoagulation + dual antiplatelet therapy (Table 1). 12.8% patients (n = 17) received anticoagulation alone due to high bleeding risk. Anticoagulant and antiplatelet medication usage showed no significant difference between MTLVA resolution and non-resolution groups (Table 1).

### 3.3 Coronary Revascularization and Medication Therapy

Among the patients, 73.7% (n = 98) underwent PCI, 19.5% (n = 26) underwent coronary artery bypass grafting (CABG) or a combination of PCI and CABG, and 6.8% (n = 9) received conservative treatment. The rates of coronary revascularization did not differ significantly between the resolution and non-resolution groups (Table 1). However, STEMI involving the left circumflex artery (LCX) was more common in the non-resolution group compared to the resolution group (45.9% vs. 27.1%, *p* = 0.026).

### 3.4 Echocardiographic Measurements

LVEF was significantly lower in the MTLVA non-resolution group compared to the MTLVA resolution group (mean 41.7% vs. 45.7%, *p* = 0.008). Additionally, the size of the LVA and MT was significantly larger in the MTLVA non-resolution group than in the MTLVA resolution group (all *p* < 0.001, Table 1).

**Fig. 1.**
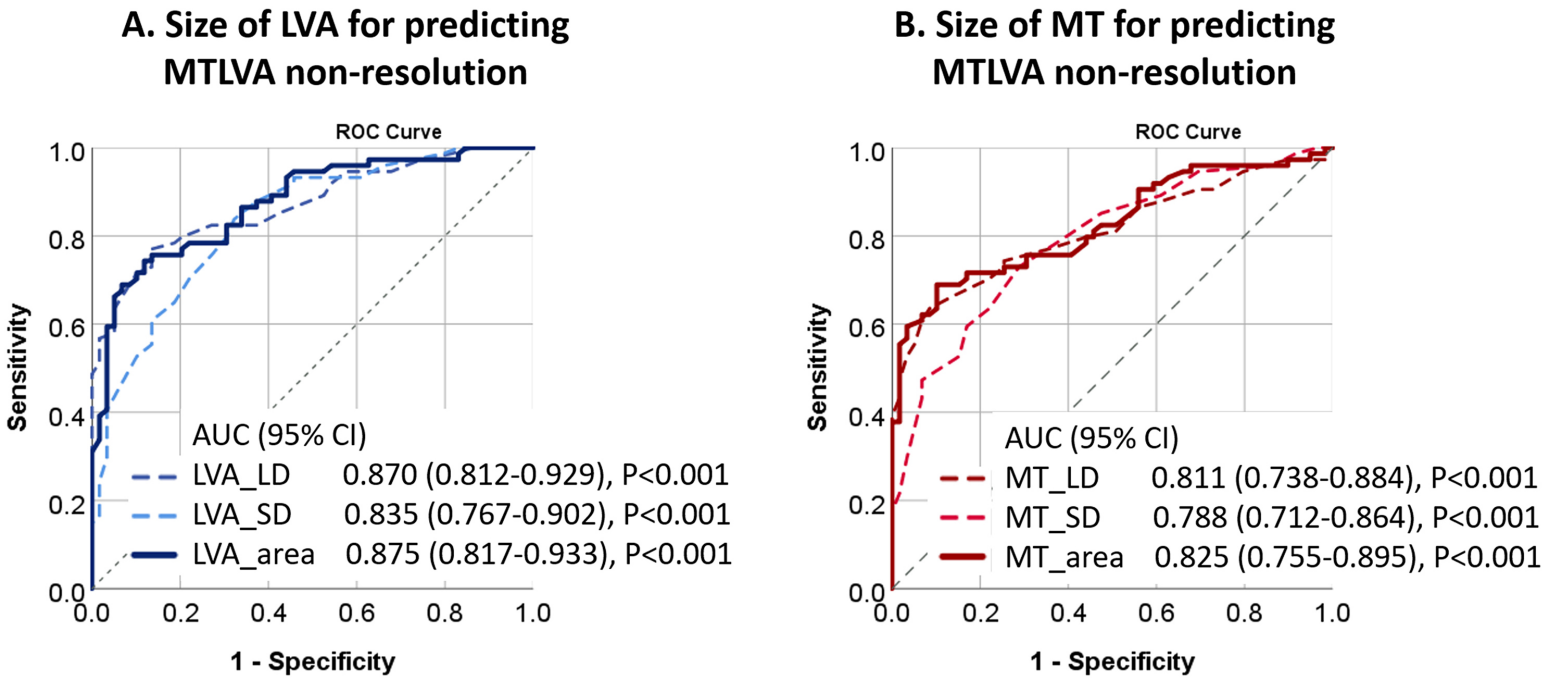
**Diagnostic performance of LVA and MT in predicting MTLVA non-resolution**. (A) ROC curve showing the diagnostic performance of LVA_area for predicting MTLVA non-resolution, with an AUC of 0.875. (B) ROC curve demonstrating the diagnostic ability of MT_area, with an AUC of 0.825, both indicating strong predictive value. CI, confidence interval; MT, mural thrombus; ROC, receiver operating characteristic; AUC, areas under curves.

**Fig. 2.**
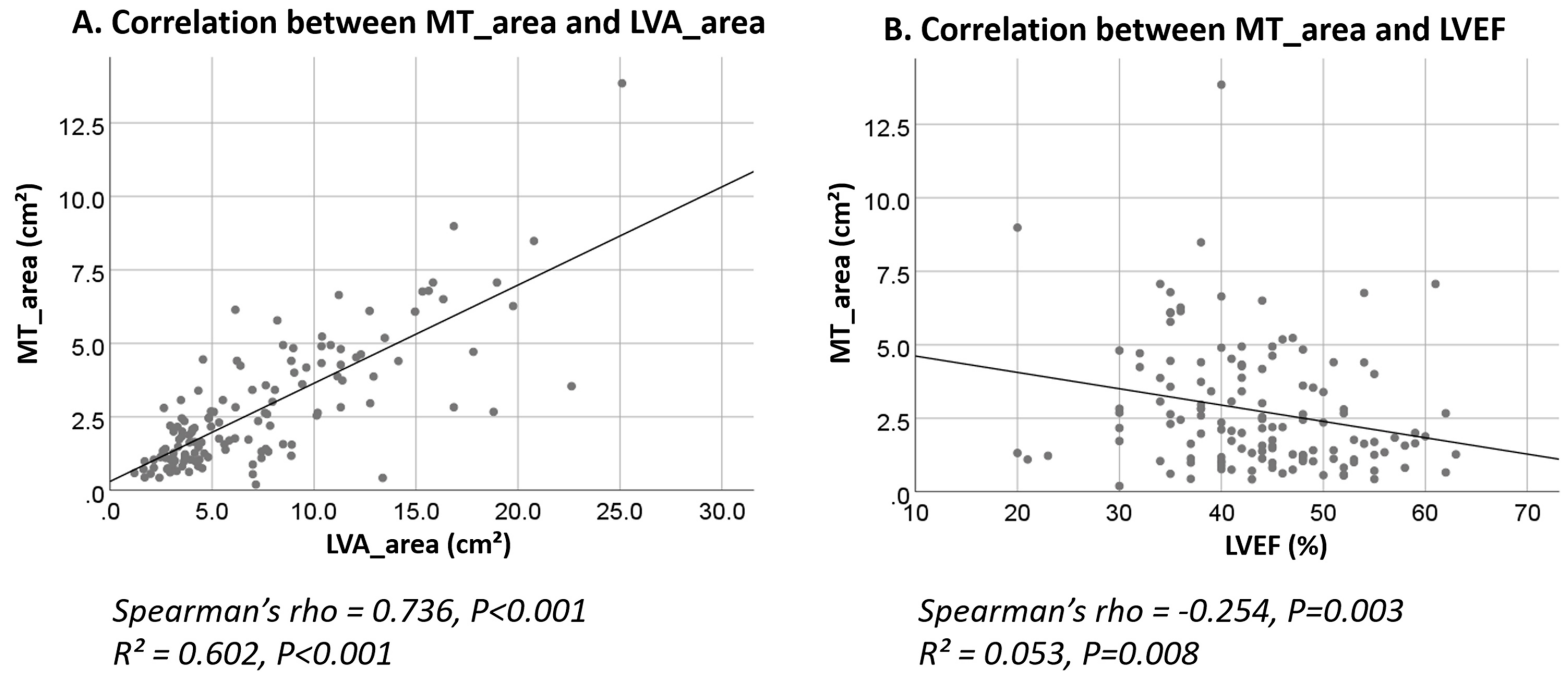
**Correlation analysis of LVA_area, MT_area, and LVEF in relation to MTLVA non-resolution**. (A) Scatter plot showing a strong positive correlation between MT_area and LVA_area, suggesting that larger thrombus and aneurysm areas are linked to MTLVA non-resolution. (B) Scatter plot showing a negative correlation between LVEF and MT_area, indicating that lower ejection fraction is associated with larger mural thrombus areas.

Larger LVA_area and MT_area demonstrated excellent diagnostic performance for predicting MTLVA non-resolution, with an AUC of 0.875 (95% CI 0.817–0.933, *p* < 0.001; Fig. 1A) and an AUC of 0.825 (95% CI 0.755–0.895, *p* < 0.001; Fig. 1B), respectively. Correlation analysis revealed a positive association between MT_area and LVA_area with MTLVA non-resolution (Spearman’s rho, *r* = 0.736, *p* < 0.001; *R*^2^ = 0.602, *p* < 0.001; Fig. 2A). LVEF exhibited a negative correlation with MT_area (Spearman’s rho, *r* = –0.254, *p* = 0.003; *R*^2^ = 0.053, *p* = 0.008; Fig. 2B).

### 3.5 Independent Risk Factors of MTLVA Non-Resolution 

Univariable Cox regression analysis identified several potential risk factors for non-resolution of MTLVA, including hypercholesterolemia, LCX-related STEMI, D-Dimer >780 ng/mL, lipoprotein (a) [Lp(a)] >270 mg/L, baseline LVEF <40%, LVA_area >4.5 cm^2^, and MT_area >2.2 cm^2^ (Table 2).

**Table 2.** **Independent risk factors associated with MTLVA non-resolution in STEMI patients with MTLVA**.

Univariable Cox model		Univariable HR (95% CI)	*p* value
	Age	0.990 (0.990–1.011)	0.338
	Male vs. female	1.327 (0.712–2.471)	0.373
	Hypercholesterolemia	1.624 (1.018–2.590)	0.042
	LCX-related STEMI	1.871 (1.175–2.978)	0.008
	D-Dimer >780 ng/mL	1.628 (1.001–2.648)	0.050
	Lp(a) >270 mg/L	1.889 (1.130–3.159)	0.015
	Baseline LVEF <40%	1.681 (1.061–2.664)	0.027
	LVA_area >4.5 cm^2^	3.544 (1.944–6.461)	<0.001
	MT_area >2.2 cm^2^	2.952 (1.779–4.900)	<0.001
Model A		^a^ Multivariable HR (95% CI)	*p* value
	Hypercholesterolemia	2.135 (1.243–3.667)	0.006
	LCX-related STEMI	2.228 (1.281–3.875)	0.005
	Lp(a) >270 mg/L	2.270 (1.315–3.918)	0.003
	LVA_area >4.5 cm^2^	4.038 (2.083–7.829)	<0.001
Model B		^a^ Multivariable HR (95% CI)	*p* value
	Hypercholesterolemia	1.715 (1.017–2.893)	0.043
	LCX related STEMI	1.875 (1.099–3.196)	0.021
	Lp(a) >270 mg/L	2.264 (1.323–3.876)	0.003
	MT_area >2.2 cm^2^	2.398 (1.377–4.178)	0.002

^a^ Multivariable Cox regression with “backward stepwise” method. Variables in the Model A included age, sex, hypercholesterolemia, LCX-related STEMI, D-Dimer >780 ng/mL, Lp(a) >270 mg/L, baseline LVEF <40%, and LVA_area >4.5 cm^2^. Variables in the Model B included age, sex, hypercholesterolemia, LCX related STEMI, D-Dimer >780 ng/mL, Lp(a) >270 mg/L, baseline LVEF <40%, and MT_area >2.2 cm^2^. HR, hazard ratio.

Given the significant collinearity between LVA_area and MT_area (Spearman’s rho = 0.736, *p* < 0.001; Fig. 2A), two separate multivariable models were constructed to address this issue and ensure the stability of the analysis. Model A included LVA_area, while Model B incorporated MT_area.

In Model A, which adjusted for age, sex, hypercholesterolemia, LCX-related STEMI, D-Dimer >780 ng/mL, Lp(a) >270 mg/L, baseline LVEF <40%, and LVA_area >4.5 cm^2^, hypercholesterolemia (HR 2.135, *p* = 0.006), LCX-related STEMI (HR 2.228, *p* = 0.005), Lp(a) >270 mg/L (HR 2.270, *p* = 0.003), and LVA_area >4.5 cm^2^ (HR 4.038, *p* < 0.001) were independently associated with non-resolution of MTLVA. Similarly, in Model B, adjusting for the same covariates, hypercholesterolemia (HR 1.715, *p* = 0.043), LCX-related STEMI (HR 1.875, *p* = 0.021), Lp(a) >270 mg/L (HR 2.264, *p* = 0.003), and MT_area >2.2 cm^2^ (HR 2.398, *p* = 0.002) remained significant predictors of non-resolution.

### 3.6 Dynamic Changes in LVEF Associated with Non-Resolution of MTLVA

**Fig. 3.**
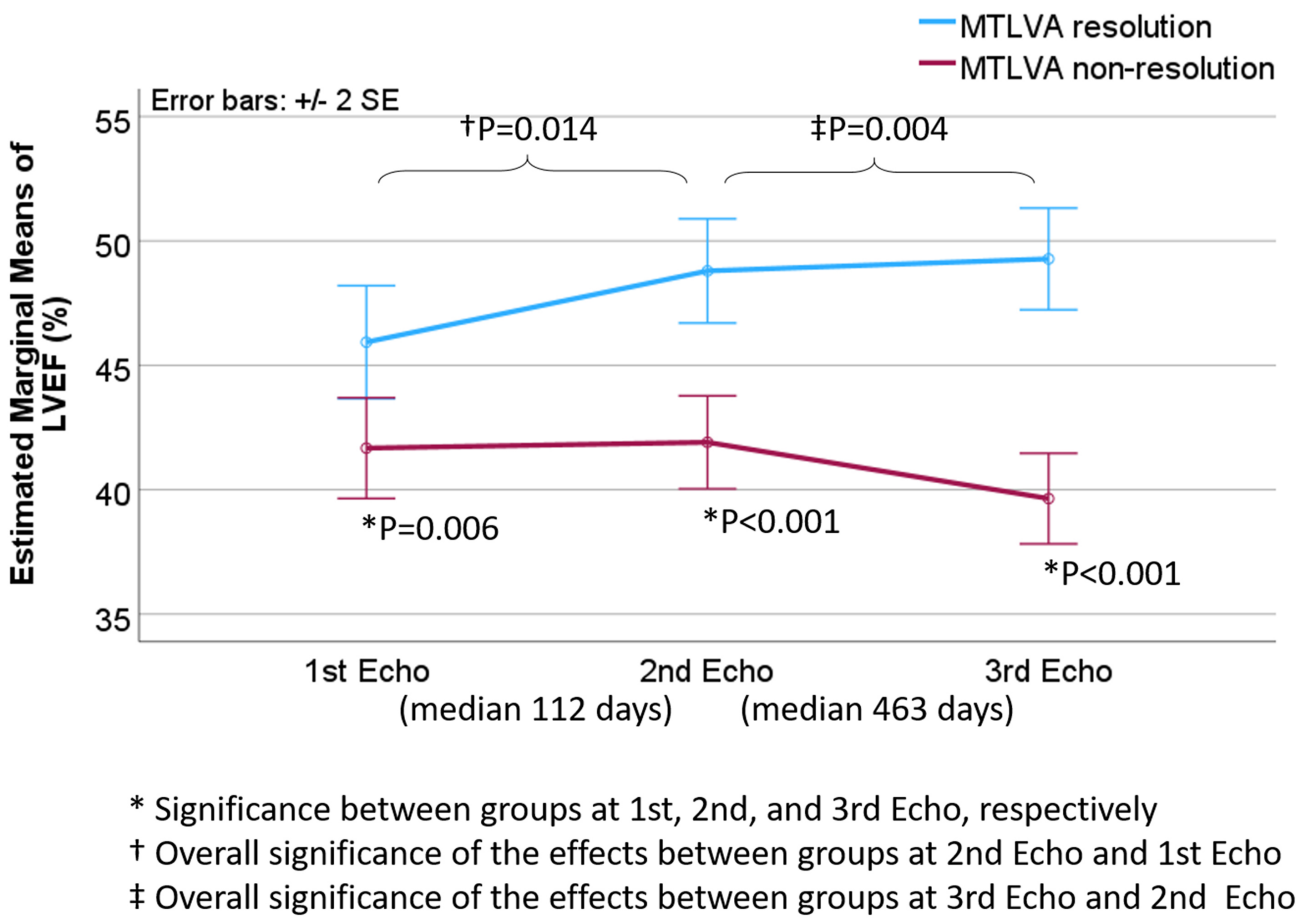
**Dynamic changes in LVEF associated with non-resolution of MTLVA**. The plot shows that, throughout the follow-up period, the mean LVEF remained significantly lower in the non-resolution group compared to the resolution group. In contrast, the resolution group exhibited a consistent increase in LVEF over time. The differences between the two groups were statistically significant at the 1st, 2nd, and 3rd echocardiographic evaluations (*p* = 0.006, <0.001, and <0.001, respectively).

As depicted in Fig. 3, throughout the follow-up periods, the mean LVEF was consistently lower in the non-resolution group, while in the resolution group, LVEF demonstrated a trend of increase over time (*p* = 0.006, <0.001, and <0.001 at the 1st, 2nd, and 3rd echocardiography examination, respectively).

### 3.7 Clinical Outcomes Associated with Non-resolution of MTLVA

**Fig. 4.**
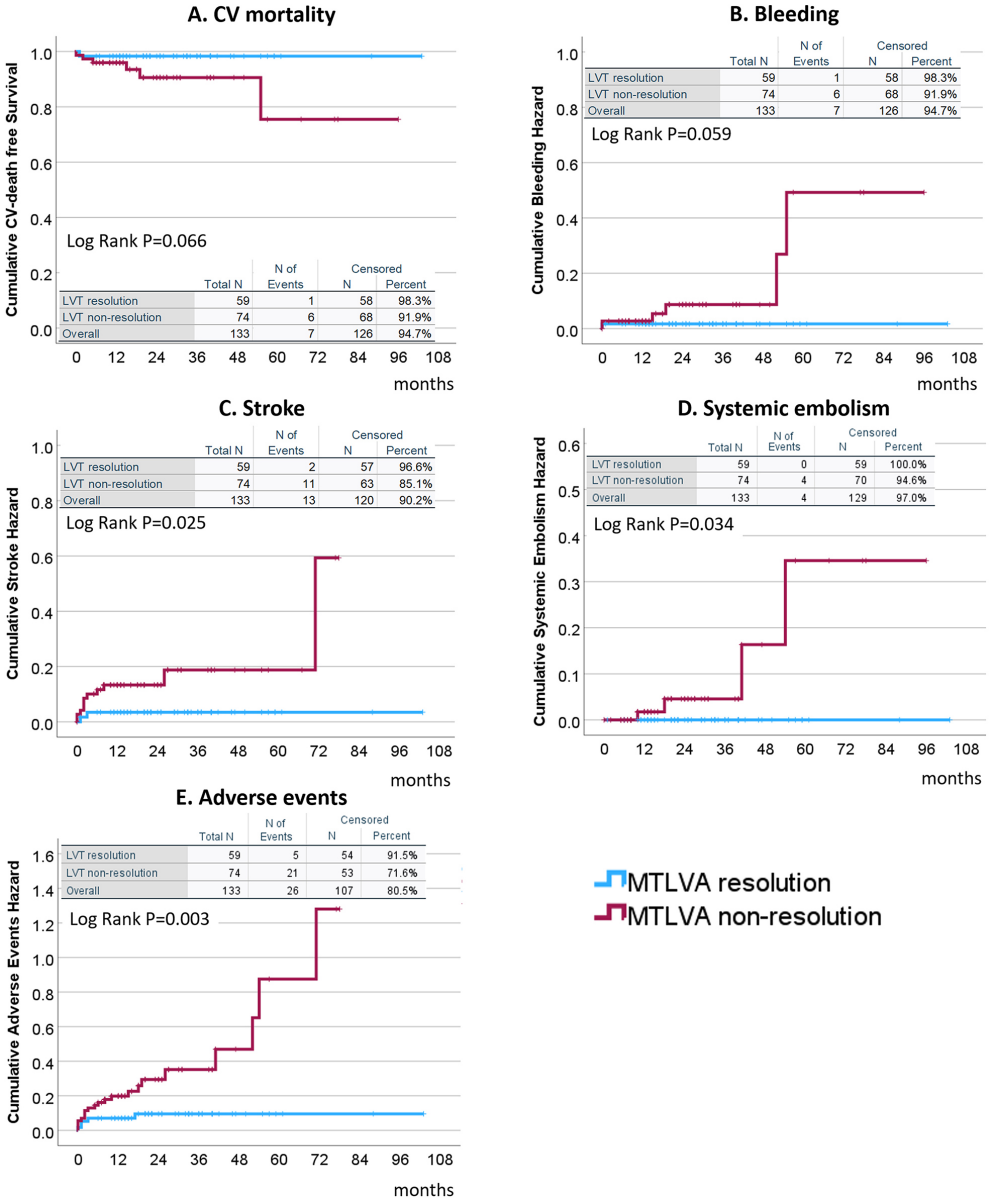
**Clinical outcomes associated with non-resolution of MTLVA**. Non-resolution of MTLVA was associated with worse clinical outcomes. Cardiovascular mortality (A) and major bleeding events (B) showed higher rates in the non-resolution group (*p* = 0.066 and *p* = 0.059, respectively). Stroke (C) was significantly more frequent in the non-resolution group (14.9% vs. 3.4%, *p* = 0.025). Systemic embolism (D) occurred in 5.4% of the non-resolution group but was absent in the resolution group (*p* = 0.034). The composite adverse event rate (E), including cardiovascular death, major bleeding, stroke, and systemic embolism, was significantly higher in the non-resolution group (28.4% vs. 8.5%, *p* = 0.003). LVT, left ventricular thrombus.

Non-resolution of MTLVA was associated with significantly worse outcomes compared to resolution (Table 1 and Fig. 4). Cardiovascular mortality (Fig. 4A) was higher in the non-resolution group (8.1% vs. 1.7%), though not statistically significant (*p* = 0.066). Major bleeding events (Fig. 4B) showed a similar trend, occurring more frequently in the non-resolution group (8.1% vs. 1.7%, *p* = 0.059). Stroke (Fig. 4C) was significantly more common in the non-resolution group (14.9% vs. 3.4%, *p* = 0.025). Systemic embolism (Fig. 4D) occurred in 5.4% of the non-resolution group but was absent in the resolution group (*p* = 0.034). The composite of adverse events (Fig. 4E), including stroke, re-revascularization, major bleeding, systemic embolism, and cardiac death, was significantly higher in the non-resolution group than in the resolution group (28.4% vs. 8.5%, *p* = 0.003).

### 3.8 Independent Risk Factors of Adverse Events

Present study identified 6 potential prognostic markers associated with the occurrence of adverse events in this cohort, including N-terminal pro-brain natriuretic peptide (NT-proBNP), serum creatinine, total cholesterol (TC), LVEF, LVA_area, and MTLVA non-resolution (Table 3). Multivariable Cox regression analysis revealed that NT-proBNP, TC, and MTLVA non-resolution remained independently associated with adverse events, while the correlation with adverse events for serum creatinine level, LVEF, and LVA_area was no longer significant after multivariable adjustment (Table 3).

**Table 3.** **Independent risk factors associated with adverse events in STEMI patients with MTLVA**.

Univariable Cox model	Univariable HR (95% CI)	*p* value
Age (years)	0.989 (0.954–1.026)	0.559
Male vs. female	5.559 (0.746–41.409)	0.094
Ln (NT-proBNP)	1.528 (1.145–2.039)	0.004
Cr (mg/dL)	1.008 (1.001–1.015)	0.029
TC (mmol/L)	0.585 (0.377–0.909)	0.017
Baseline LVEF (%)	0.936 (0.892–0.983)	0.008
LVA_area (cm^2^)	1.072 (0.995–1.172)	0.066
MTLVA non-resolution	3.314 (1.226–8.959)	0.018
Multivariable Cox model	^a^ Multivariable HR (95% CI)	*p* value
Ln (NT-proBNP)	1.460 (1.103–1.933)	0.008
TC (mmol/L)	0.648 (0.408–1.029)	0.066
MTLVA non-resolution	3.270 (1.077–9.930)	0.037

^a^ Multivariable Cox regression with “backward stepwise” method (Likelihood ratio). Variables in the multivariable model included age, sex, Ln (NT-proBNP), Cr, TC, baseline LVEF, and MTLVA non-resolution.

## 4. Discussion

This retrospective study aimed to identify independent risk factors for the non-resolution of MT in patients with LVA following STEMI. Our analysis revealed that a significant proportion of patients with MTLVA (55.6%) did not experience thrombus resolution. Key independent clinical predictors of non-resolution included elevated lipoprotein (a) [Lp(a)] levels (>270 mg/L), larger LVA area (>4.5 cm^2^), and greater MT area (>2.2 cm^2^), beyond other established risk factors such as hypercholesterolemia and LCX-related STEMI. Additionally, a negative correlation was observed between LVEF and thrombus area, with patients in the non-resolution group having lower baseline LVEF and less improvement during follow-up compared to the resolution group. The non-resolution group also demonstrated a higher incidence of adverse clinical outcomes, including stroke, re-revascularization, major bleeding, systemic embolism, and cardiac death (28.4% vs. 8.5%, *p* = 0.003).

### 4.1 Thrombus Regression and Suboptimal Anticoagulation Therapy

Our study highlights the ongoing challenge of MTLVA non-resolution despite contemporary antithrombotic therapies. In our cohort, resolution occurred in only 45.4% of STEMI patients with MTLVA over a median follow-up of 25 months. Similarly, Lattuca *et al*. [5] reported a resolution rate of 62.3% within 103 days, while Zhou *et al*. [4] observed non-resolution in 32.6% of patients during a median follow-up of 1.2 years, with 24.3% experiencing recurrence.

Importantly, our study identified independent predictors of MTLVA non-resolution, including elevated Lp(a) levels (>270 mg/L), larger LVA area (>4.5 cm^2^), and greater MT area (>2.2 cm^2^). These findings underline the clinical importance of risk stratification. Furthermore, patients with non-resolution consistently exhibited lower LVEF than those with resolution throughout follow-up, while the latter group showed progressive LVEF improvement, suggesting a relationship between thrombus resolution and myocardial recovery.

We observed that a higher proportion of patients in the resolution group were on VKAs compared to the non-resolution group (66.1% vs. 50.0%, *p* = 0.062). In contrast, the non-resolution group had a greater proportion of patients on non-vitamin K oral anticoagulants (NOACs, 50.0% vs. 28.8%, *p* = 0.013). This suggests that VKAs may be more effective in facilitating thrombus resolution compared to NOACs in this patient cohort with MT in the LVA. However, previous studies, although based on small sample sizes, have generally reported similar LV thrombus resolution rates between VKAs and NOACs [14]. Therefore, the comparative efficacy of VKAs versus NOACs in thrombus resolution should be explored in larger, prospective studies to better understand their roles in this clinical context.

### 4.2 Lower LVEF and Risk of Thrombus Non-Resolution

Our analysis revealed that a baseline LVEF <40% was a potential risk factor associated with non-resolution of MTLVA in this cohort. Previous studies have indicated that patients with LVEF <40% are at an increased risk of developing LVT due to blood stasis in the ventricular cavity and endocardial injury with associated inflammation [15]. Allard *et al*. [16] demonstrated that lower LVEF was predictive of LVT. Our study cohort further showed that LVEF was significantly lower in the MTLVA non-resolution group compared to the MTLVA resolution group (mean 41.7% vs. 45.7%, *p* = 0.008). Additionally, patients with persistently lower LVEF over time also exhibited an increased risk of MTLVA non-resolution.

### 4.3 Higher Levels of Lp(a) and D-Dimer Level and Thrombus Non-Resolution

Lp(a) is a genetically determined lipoprotein with pro-thrombotic and anti-fibrinolytic properties [17], recognized as a causal risk factor for atherosclerotic cardiovascular disease and associated with vascular inflammation and thrombus burden [18, 19, 20, 21, 22, 23]. While Celik *et al*. [24] reported no association between Lp(a) and LVT risk in acute myocardial infarction, our findings suggest that Lp(a) >270 mg/L is independently associated with an increased risk of MTLVA non-resolution. This association may reflect the pro-thrombotic role of Lp(a) in MTLVA pathogenesis, but further studies are needed to clarify this relationship.

D-dimer, a marker of coagulation and fibrinolysis activation in response to the body’s hypercoagulable state [25, 26], has been associated with thrombus formation in conditions like dilated cardiomyopathy and post-myocardial infarction (MI) LV dysfunction [27, 28]. Although we observed a potential link between elevated D-dimer levels (>780 ng/mL) and MTLVA non-resolution, this association lost significance after adjustment. Future studies are required to determine whether D-dimer could reliably predict MTLVA non-resolution.

### 4.4 Larger LVA_area and MT_area and Thrombus Non-Resolution

Patients with an LVA_area >4.5 cm^2^ had a nearly 4-fold higher risk of MTLVA non-resolution compared to those with an LVA_area ≤4.5 cm^2^. Similarly, patients with an MT_area >2.2 cm^2^ faced approximately a 2-fold increased risk of MTLVA non-resolution compared to those with an MT_area ≤2.2 cm.

The association between larger LVA_area and MT_area with thrombus non-resolution remains unclear. Several factors may contribute to this relationship: (1) In larger LVA areas, blood flow could be slower, potentially resulting in a more stable thrombus that is less susceptible to resolution; (2) Larger thrombus areas might be more resistant to effective resolution by antithrombotic therapy, possibly due to insufficient drug penetration or thrombus composition. Given these considerations, it may be reasonable to extend the duration of antithrombotic therapy in patients with larger LVA_area and MT_area to improve the chances of thrombus resolution.

### 4.5 Severe Myocardial Injury and Reperfusion Injury as Contributors to Thrombus Non-Resolution

Severe myocardial injury, especially in large infarctions, creates a pro-thrombotic environment by promoting persistent endothelial dysfunction, local inflammation, and microvascular damage [29]. These pathological processes facilitate MT formation and hinder its resolution. In our study, patients with larger LVA areas (LVA_area >4.5 cm^2^) and MT areas (MT_area >2.2 cm^2^) were at significantly higher risk of thrombus non-resolution. These findings underscore the role of extensive myocardial damage as a sustained substrate for thrombus persistence.

Reperfusion injury adds another layer of complexity. The restoration of blood flow to ischemic myocardium, while critical for salvaging viable tissue, can intensify damage through microvascular obstruction, oxidative stress, and inflammation. This cascade is often accompanied by intramyocardial hemorrhage (IMH), a result of microvascular rupture during reperfusion [30, 31]. IMH introduces blood products into the myocardium, forming a nidus for thrombus development and further complicating its resolution. The recent Canadian Society of Cardiology definition of tissue injury severity emphasizes the impact of extensive myocardial injury, including IMH, on adverse clinical outcomes [31], and highlights the interplay between severe myocardial injury and reperfusion injury in driving thrombus persistence. Together, these mechanisms suggest that even under standard antithrombotic therapy, severe tissue damage and associated complications like IMH play a pivotal role in the non-resolution of MT. Future studies utilizing advanced imaging modalities, such as cardiac magnetic resonance imaging, could provide a deeper understanding of these mechanisms. Such insights may help identify high-risk patients and inform tailored therapeutic strategies to improve thrombus resolution and clinical outcomes.

### 4.6 Non-Resolution of MTLVA and Clinical Outcomes

Our results confirm that MTLVA non-resolution is associated with worse clinical outcomes. Non-resolution patients in our study had a higher incidence of adverse events compared to resolution patients. Consistent with Zhou *et al*. [4], who reported higher rates of MACE, embolic events, and stroke among patients with LVT recurrence, these findings underscore the importance of achieving complete thrombus resolution to reduce adverse cardiovascular and cerebrovascular outcomes.

### 4.7 Clinical Implication 

Defining independent risk factors of non-resolution of MTLVA might add the risk stratification of patients with MTLVA. Patients with higher risk of non-resolution MTLVA might benefit from more guideline-adherent antithrombotic treatment.

### 4.8 Study Limitations

This study has several limitations. First, its retrospective design and single-center setting introduce the possibility of selection bias, which may limit the generalizability of our findings. Larger and prospective studies are needed to confirm these results and provide more robust evidence. Second, the diagnosis of MTLVA was primarily based on transthoracic echocardiography, which, while a standard and widely accessible imaging modality, may lack the sensitivity and specificity of advanced techniques such as contrast-enhanced ultrasound or cardiac magnetic resonance imaging. Future studies incorporating these modalities could enhance diagnostic accuracy and provide additional insights. Third, we did not capture reperfusion metrics such as symptom-to-balloon or door-to-balloon times, which are critical determinants of STEMI outcomes and may have influenced our findings. Finally, the relatively small sample size, although representative of this patient population, may have reduced the statistical power to detect certain associations. Thus, our findings should be interpreted as exploratory and hypothesis-generating, warranting validation in larger, multicenter cohorts.

## 5. Conclusions

In STEMI patients with MTLVA, non-resolution of mural thrombus is associated with a significantly higher risk of adverse events, including stroke and systemic embolism, compared to those with thrombus resolution. Independent risk factors for non-resolution include elevated Lp(a) levels, larger LVA and MT areas. These factors underscore the complexity of thrombus resolution and highlight the need for personalized management strategies. Prolonged or intensified antithrombotic therapy may benefit patients with non-resolution MTLVA. An individualized risk stratification approach, considering patient characteristics, disease vessel features, and thrombus evolution, should guide clinical decision-making and improve outcomes in this patient population.

## Data Availability

Data are available on reasonable request (contact the corresponding author Dr. Junhua Ge).

## Abbreviations

ALT, alanine aminotransferase; AST, aspartate aminotransferase; BMI, body mass index; BUN, blood urea nitrogen; CABG, coronary artery bypass grafting; CI, confidence interval; CRP, C-reactive protein; Cr, creatinine; DAPT, dual antiplatelet therapy; HR, hazard ratio; IMH, intramyocardial hemorrhage; LAD, left anterior artery; LCX, left circumflex artery; LDL-C, low-density lipoprotein cholesterol; Lp(a), lipoprotein(a); LVA, left ventricular aneurysm; LVA_LD, left ventricular aneurysm long diameter; LVA_SD, left ventricular aneurysm short diameter; LVEF, left ventricular ejection fraction; MACE, major adverse cardiovascular events; MT, mural thrombus; MT_LD, mural thrombus long diameter; MT_SD, mural thrombus short diameter; MTLVA, mural thrombus in left ventricular aneurysm; NOACs, non-vitamin K oral anticoagulants; NT-proBNP, N-terminal pro-brain natriuretic peptide; PCI, percutaneous coronary intervention; RCA, right coronary artery; STEMI, ST-segment elevation myocardial infarction; TC, total cholesterol; TTE, transthoracic echocardiography; TG, triglyceride; UA, uric acid; VKAs, vitamin K antagonists.
